# HiRNet: A Hierarchical-Refinement-Guided Hybrid Retinal Vessel Segmentation Network for Smartphone-Based Fundus Images

**DOI:** 10.3390/s26196218

**Published:** 2026-09-30

**Authors:** Yuqing Yin, Liang Niu, Suyan Li, Yifan Mu, Xiao Xu, Yan Wang

**Affiliations:** 1 School of Computer Science and Technology/School of Artificial Intelligence, China University of Mining and Technology, Xuzhou 221116, China; yinyuqing@cumt.edu.cn (Y.Y.);; 2 School of Information and Control Engineering, China University of Mining and Technology, Xuzhou 221116, China; 3 Nankai University Eye Institute, Nankai University, Tianjin 300350, China

**Keywords:** smartphone-based, hierarchical refinement, hybrid network, hierarchical context, multi-path attention

## Abstract

Smartphone-based fundus photography provides a low-cost and convenient approach to retinal screening and follow-up examinations. As a fundamental step in fundus image analysis, retinal vessel segmentation plays an important role in the diagnosis and monitoring of retinal diseases. However, existing methods often exhibit limited performance on smartphone-based fundus images. This limitation mainly arises from the significantly lower image quality that affects the clarity and detail of the retinal vessels, making accurate segmentation more difficult. To address these challenges, we propose a hierarchical-refinement-guided hybrid network (HiRNet), which incorporates a hybrid CNN–Transformer feature extractor to jointly model local vascular structures and global contextual information. Specifically, consecutive asymmetric dilated convolutions capture multi-scale local features, while multi-path dilated attention enhances contextual representations across different receptive fields. In addition, a hierarchical context information transmission module is introduced to progressively integrate features from different resolutions during multistage upsampling, thereby improving vessel continuity and boundary delineation. Experiments on RVD, DRIVE, and CHASE_DB1 demonstrate the effectiveness of HiRNet. On the smartphone-based RVD dataset, HiRNet obtains the highest mean sensitivity (58.36%), specificity (97.81%), accuracy (95.83%), F1-score (58.39%), and AUC (95.16%). The improvements in sensitivity, accuracy, F1, and AUC over the strongest competing methods are statistically significant. HiRNet also obtains sensitivity values of 82.73% and 82.13%, accuracy values of 95.59% and 95.98%, F1-scores of 82.76% and 80.37%, and AUC values of 98.83% and 97.90% on DRIVE and CHASE_DB1, respectively. These results indicate that HiRNet provides a relative improvement on the challenging domain of smartphone-based fundus images while maintaining strong performance on conventional fundus datasets.

## 1. Introduction

**Figure 1:**
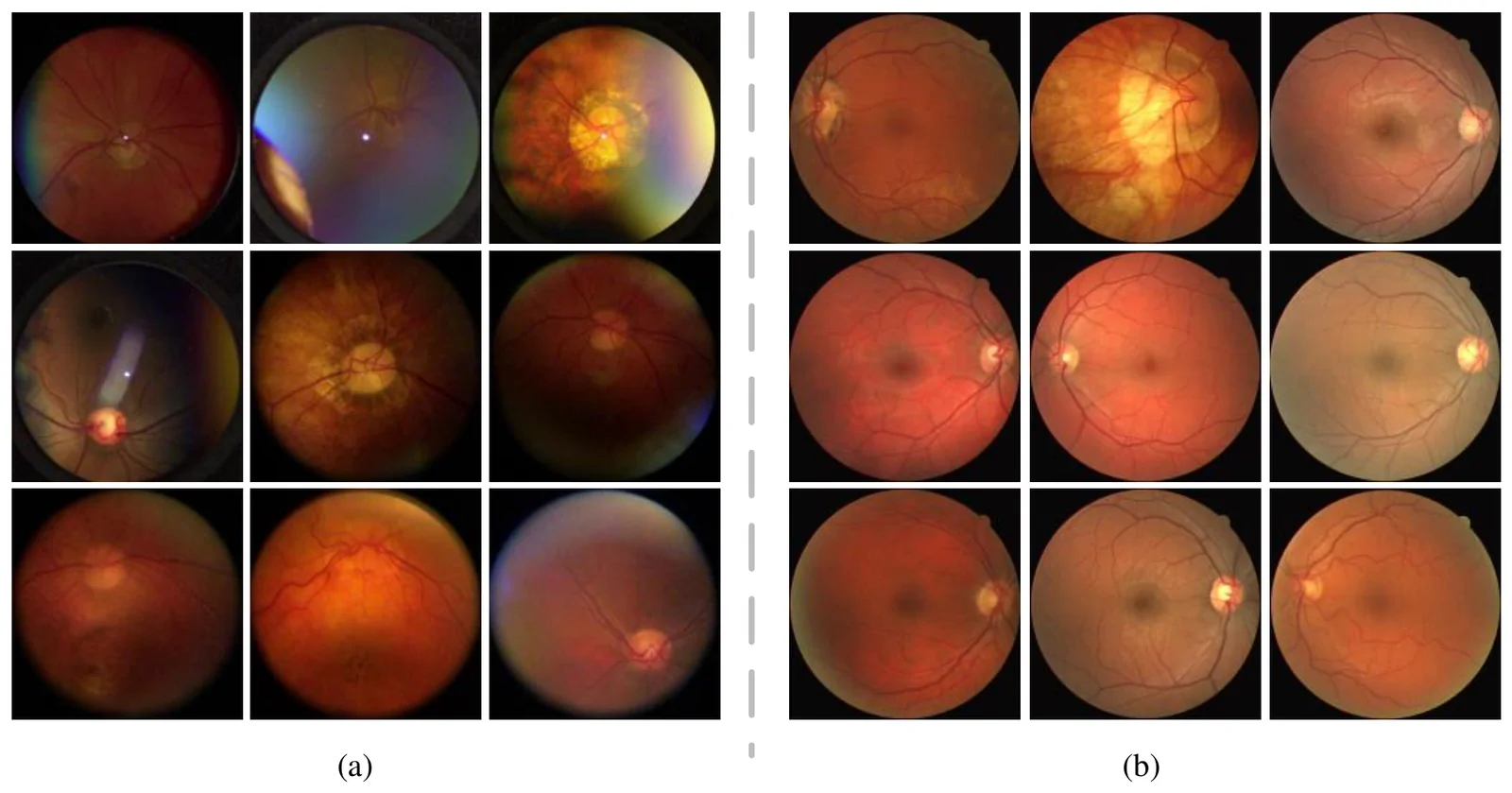
Fundus images photographed by different devices. (**a**) Smartphone-based devices. (**b**) Bench-top devices.

Smartphone-based fundus photography has proven effective for retinal screening and follow-up examinations [1,2,3]. It provides a more accessible and cost-effective approach to the early detection, prognostic assessment, and treatment monitoring of various ocular and systemic diseases, including glaucoma, diabetic retinopathy, and arteriosclerosis. However, smartphone-based fundus images often exhibit lower and more variable image quality. Figure 1a and Figure 1b show fundus images acquired using smartphone-based devices and bench-top devices, respectively. Although smartphone-based devices may produce images with a high nominal resolution, their simplified optics systems and nonuniform illumination frequently degrade image quality, disrupting vessel continuity and reducing the contrast of small vascular branches. These deficiencies significantly hinder the accurate analysis of the retinal vasculature and pose a major barrier to the quantification of vascular biomarkers in widespread adoption of smartphone-based fundus photography.

Recently, fundus image segmentation has made significant progress and achieved impressive performance [4,5,6,7]. According to the available annotated data, most existing methods can be categorized as unsupervised methods [8] or supervised methods [9,10,11]. The unsupervised methods mainly extract structure by distinguishing vascular structures from the surrounding background [12]. Due to the lack of detailed supervision, these methods often face challenges in detecting fine structures such as blood vessels. To achieve better performance, supervised methods such as U-Net and its variants have been proposed to aggregate fine-grained features generated by the encoder and decoder through skip connections [13,14]. Although these methods have substantially improved segmentation performance, they are primarily designed for bench-top fundus images and have yet to fully consider the unique characteristics of smartphone-based fundus images. On the one hand, vascular branches in smartphone-based images are often poorly defined and difficult to distinguish. This exacerbates the challenge of accurately segmenting fine vessel structures, as repeated convolution and pooling operations can provide powerful feature aggregation capability but unfortunately deteriorate spatial information and detail in raw images [15,16,17]. On the other hand, the simple skip connections often transfer feature information from encoder layers [18], leading to boundary inaccuracies in the final segmentation.

To address these issues, we propose a novel segmentation network for smartphone-based fundus images, termed the hierarchical-refinement-guided hybrid retinal vessel segmentation network (HiRNet). Considering the feature misalignment between convolutional neural networks (CNNs) and transformer, the hybrid CNN–Transformer feature extractor (HCTFE) is designed to obtain complementary local and global feature representations. In each stage of the encoder, the consecutive asymmetric dilated convolution (CADC) module is adopted to capture enhanced multi-scale local features, and the multi-path dilated transformer (MPDT) is used to perform self-attention and capture long-range contextual dependencies within the dilated attention field. Then, the hierarchical features produced by current HCTFE are concatenated and fused to serve as the input for the next stage. Finally, a hierarchical context information transmission (HCIT) is proposed as an alternative to conventional upsampling methods, aggregating hierarchical features by leveraging the complementary strengths of high-resolution and low-resolution feature maps in the decoder stage. Overall, the proposed HiRNet focuses on improving feature representation fidelity across varying scales and guiding refined segmentation results. The main contributions are as follows:

- We propose a novel network with a focus on exploring local connectivity and global contextual information in smartphone-based fundus images. This represents an effort in the critical application of vessel segmentation using smartphone-based color fundus images.
- We propose a hierarchical information transmission module that aggregates feature maps at different resolutions to refine mask boundaries, thereby preserving fine vascular structures.
- The proposed method achieves superior retinal vessel segmentation performance on the smartphone-based fundus dataset.

## 2. Related Work

### 2.1. U-Shaped Architecture for Medical Image Segmentation

U-shaped architectures have become widely used in medical image segmentation owing to their effective integration of semantic context and spatial detail [19,20,21]. U-Net [13] established the foundation of this network family by employing a symmetric contracting–expanding architecture and transferring high-resolution encoder features to the corresponding decoder stages through skip connections. This design compensates for spatial information lost during downsampling and facilitates the recovery of object boundaries and fine anatomical structures. Subsequent studies have extended the U-shaped architecture from the perspectives of feature selection, multi-scale fusion, and representation refinement. Attention U-Net [22] incorporated attention gates into the skip pathways to emphasize task-relevant regions while suppressing responses from irrelevant background areas. Instead of using static attention, Man et al. [23] formulated region localization as a sequential decision-making process and employed deep reinforcement learning to guide the network toward informative regions. For curvilinear structure segmentation, Mou et al. [24] introduced CS-Net, which jointly models channel-wise and spatial relationships to integrate local appearance information with broader contextual information. To alleviate the semantic discrepancy between encoder and decoder features, Zhou et al. [18] replaced direct skip connections with nested and densely connected pathways, thereby enabling more effective multi-scale feature aggregation. Alom et al. [25] further embedded recurrent residual units into the U-shaped framework, allowing feature representations to be iteratively refined while improving the optimization of deeper networks. U-shaped networks have also been extended from two-dimensional images to volumetric medical data. The 3D U-Net [26] replaced two-dimensional operators with their three-dimensional counterparts, enabling end-to-end learning from volumetric inputs. V-Net [27] adopted volumetric convolutional operations together with a Dice-based objective to alleviate the class imbalance commonly encountered in medical image segmentation.

Despite these architectural advances, most U-shaped networks still rely predominantly on convolutional operations. The locality-oriented inductive bias of convolution makes it difficult to directly establish interactions between spatially distant but semantically related regions. Consequently, conventional U-shaped networks may provide insufficient global context modeling, particularly when segmenting structures characterized by long-range continuity, complex topology, or weak local contrast.

### 2.2. Vision Transformer for Medical Image Segmentation

Vision Transformers have demonstrated strong capabilities in a wide range of computer vision tasks, particularly in modeling dependencies across distant spatial regions. Motivated by this advantage, an increasing number of studies have introduced Transformer architectures into medical image segmentation. For instance, Cao et al. [19] employed a U-shaped architecture constructed with hierarchical Swin Transformer blocks, allowing the network to capture contextual information at different spatial scales. TransAttUnet [28] combined multilevel guided attention with multi-scale skip connections to improve feature representation and spatial information fusion. Lin et al. [29] incorporated hierarchical Swin Transformer blocks into both the encoder and decoder of a standard U-shaped network to learn structural representations at the pixel level. Transformer-based encoders have also been combined with task-specific feature aggregation modules. Dong et al. [30] introduced cascaded fusion, camouflage identification, and similarity aggregation modules to integrate multilevel features and distinguish inconspicuous target regions. These designs exploit the global modeling capability of Transformers while retaining multi-scale information required for dense prediction. Transformer architectures have further been extended to volumetric medical image segmentation. UNETR [31] represents a three-dimensional input volume as a sequence of image patches and employs a Transformer encoder to learn global representations, which are progressively integrated with a convolutional decoder for voxel-level prediction. UNETR++ [32] improves this structure through paired attention that jointly captures spatial and channel-wise feature relationships.

Despite their strong global representation capability, Transformer-based networks generally provide fewer inherent assumptions about spatial locality than convolutional networks. Their performance may therefore depend heavily on the scale and diversity of the available training data. This limitation is particularly relevant to medical imaging, where densely annotated datasets are often small. Moreover, insufficient emphasis on local patterns may hinder the accurate delineation of subtle structures and weak boundaries. These observations motivate the integration of convolutional feature extraction with Transformer-based global context modeling.

### 2.3. Retinal Vessel Segmentation

Retinal vessel segmentation is a crucial task in medical image segmentation as it provides essential information for diagnosing and monitoring various ocular and systemic diseases. The vascular structures targeted for segmentation typically exhibit characteristics such as thinness, continuity, and morphological complexity, often accompanied by low edge contrast. These attributes present significant challenges to segmentation tasks and highlight the need for specialized methods to improve segmentation performance. Considering the boundary detection problem in retinal vessel segmentation, Fu et al. [9] proposed an attention-guided network that aims to preserve structural information and utilize expanding operations to enhance boundary delineation. Sathananthavathi et al. [33] modified the U-Net architecture by using dilated convolutions and equalizing kernel numbers across encoder-decoder stages. In each encoder stage, two extra layers are added to improve vessel extraction and segmentation accuracy. Wu et al. [34] proposed a convolutional network comprising a backbone network and branch network. The backbone network is utilized for multi-scale feature extraction, while the branch network is responsible for vessel segmentation mask generation. Wang et al. [35] utilized Mahalanobis distance to create a highly nonlinear decision boundary. Furthermore, the cascade framework is trained through a single feed-forward pass, allowing nonlinearity to be determined by data complexity rather than predefined parameters. Qu et al. [36] incorporated a vascular weight map and a boundary loss function derived from the distance map, which places maximum weight at the vascular edges to encourage the model to focus more on critical areas. Zhou et al. [37] proposed a multi-scale context dense aggregation network to extract the blood vessel boundaries from concentric patches with different scales. More recently, researchers also proposed some other retinal vessel segmentation methods, e.g., self-supervised method [14], graph neural network-based method [38], multimodal method [39], deep matched-filtering-based method [40] and multi-scale feature-fusion-based method [41]. Despite their success, these methods were designed for bench-top fundus segmentation and remain limited when applied to smartphone-based fundus images.

## 3. Methodology

### 3.1. Framework Overview

**Figure 2:**
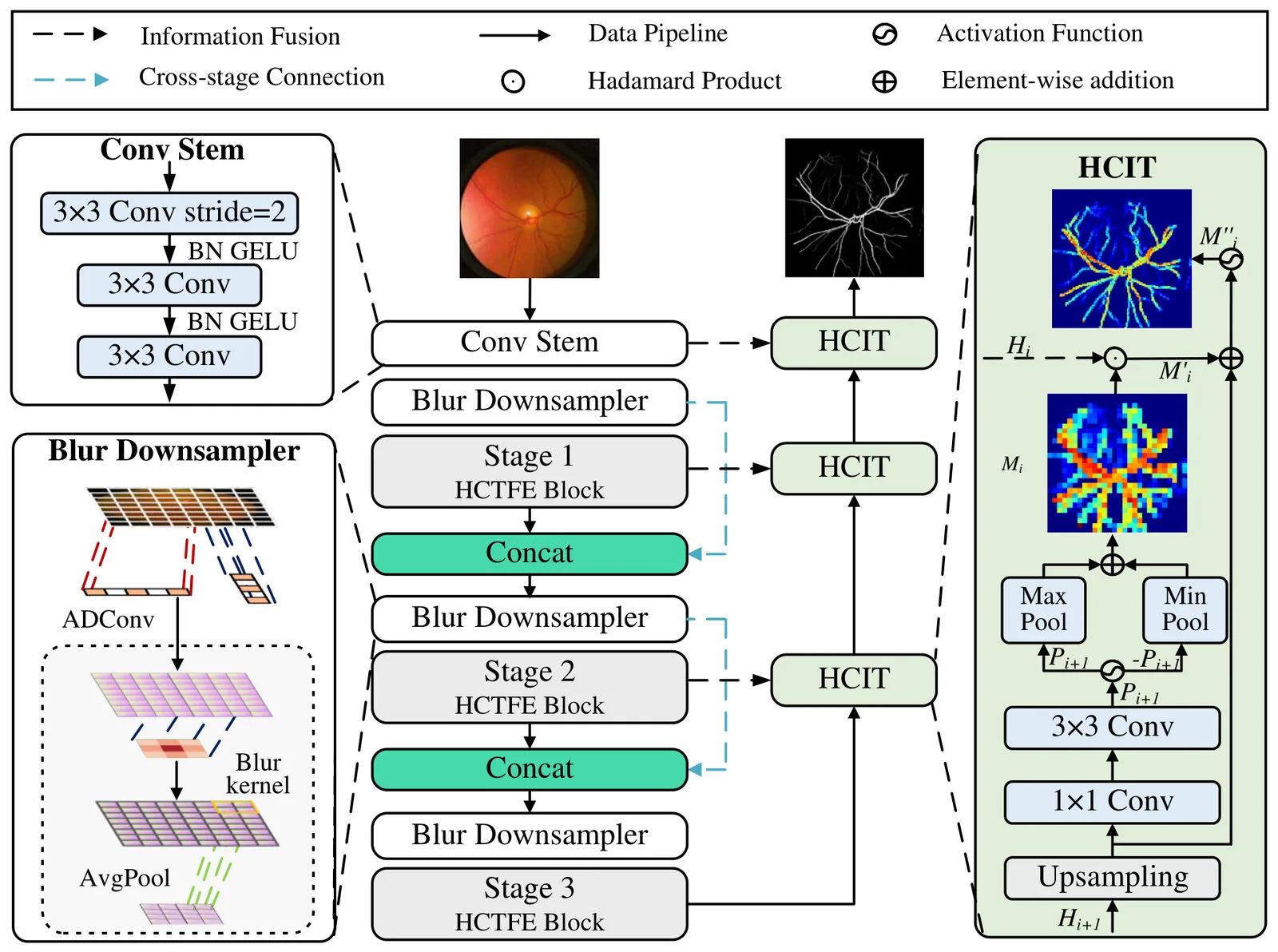
Overall architecture of HiRNet.

The overall architecture of HiRNet is illustrated in Figure 2. It follows an encoder–decoder structure, in which the encoder progressively extracts hierarchical representations through three stages. Given an RGB fundus image $X\in R^{H\times W\times 3}$, a convolutional stem [42] is first employed to capture shallow visual features. The stem comprises a 3×3 convolution with a stride of 2, followed by two 3×3 convolutions with a stride of 1, producing a feature map of size $\frac{H}{2}\times \frac{W}{2}\times 64$. A blur downsampler is subsequently applied to reduce the spatial resolution to $\frac{H}{4}\times \frac{W}{4}\times 64$ while suppressing aliasing artifacts introduced by conventional downsampling. The resulting feature map is processed by the first stage of the hybrid CNN–Transformer feature extractor. In the subsequent stages, features retained from the preceding downsampling pathway are propagated through concatenation before further transformation. This cross-stage information flow promotes feature reuse and preserves representations learned at earlier resolutions. The second and third encoder stages progressively generate feature maps of sizes $\frac{H}{8}\times \frac{W}{8}\times 128$ and $\frac{H}{16}\times \frac{W}{16}\times 192$, respectively. The decoder employs the hierarchical context information transmission module to progressively restore spatial resolution and aggregate complementary information across different feature levels. By combining high-level contextual representations with spatially detailed features, the decoder produces more precise vessel predictions and improves the delineation of vessel boundaries.

### 3.2. Blur Downsampler and Hybrid CNN–Transformer Feature Extractor

Although convolution operations can capture structural information and local features, they are likely to ignore global contextual relationships. Additionally, CNNs process image regions using shared weights, and this inductive bias makes CNNs more dependent on texture than on shape. Thus, we design a hybrid CNN–Transformer feature extractor to combine local connectivity with global contextual modeling.

#### 3.2.1. Blur Downsampler

A potential concern with downsampling is that overly aggressive filtering may lead to a loss of information. Inspired by anti-aliased pooling [43], we introduce a blur downsampler, which combines a blur kernel with standard convolution to alleviate the above limitation. As shown in Figure 2, it extracts features using a standard convolution and decreases the spatial dimension using a Gaussian filter and average pooling, which is defined as:

(1) $$f_{down}\left(X\right)=\mathrm{AvgPool}\left({\mathrm{Blur}}_{4\times 4}\left(\mathrm{GELU}\left({\mathrm{Conv}}_{3\times 3}\left(X\right)\right)\right)\right)$$

where ${\mathrm{Conv}}_{3\times 3}$ is a 3×3 standard convolution, followed by the GELU function. ${\mathrm{Blur}}_{4\times 4}$ is a Gaussian filter with size 4×4, and AvgPool(·) is average pooling. Naturally, the high-frequency noise and details in the image can be effectively reduced, which helps to reduce aliasing artifacts during the downsampling procedure.

#### 3.2.2. Consecutive Asymmetric Dilated Convolution

**Figure 3:**
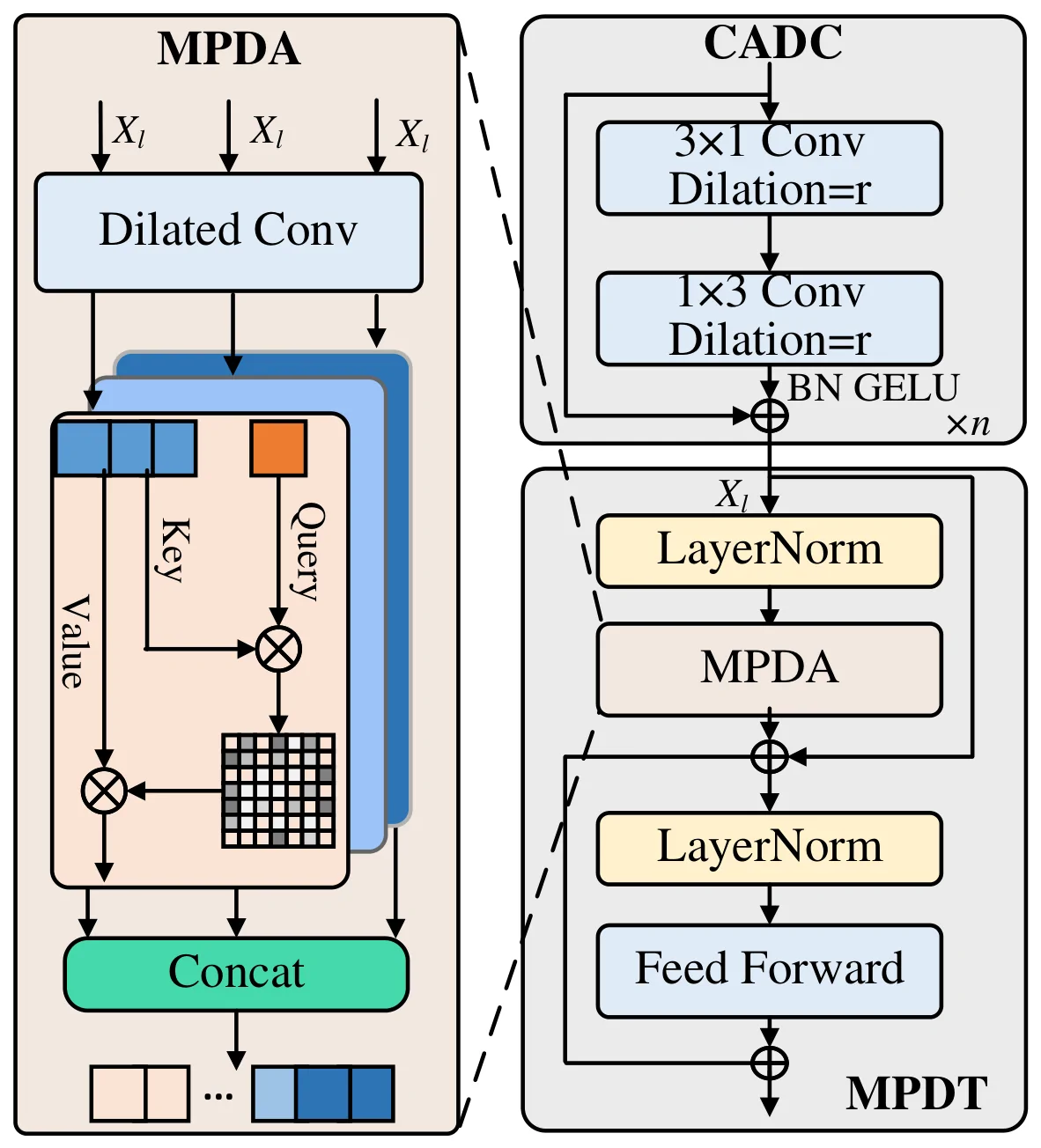
Structures of the proposed consecutive asymmetric dilated convolution (CADC) and multi-path dilated transformer (MPDT) modules. Each encoder stage contains CADC and MPDT as two separate components. The MPDT module employs multi-path dilated attention (MPDA), which contains three parallel paths with dilation rates of 1, 2, and 3.

Different from methods that employ parallel dilated convolutions only at the final network layer, we incorporate a sequence of asymmetric dilated convolutions into each encoder stage to aggregate multi-scale contextual information. As illustrated in Figure 3, each ADConv adopts an asymmetric kernel length of k=3. Specifically, an ADConv first applies a vertical 3×1 convolution and then a horizontal 1×3 convolution. Both convolutions use the same dilation rate *r*. The dilated convolution enlarges the receptive field without changing the spatial resolution of the output feature map.

For the *l*-th ADConv block, the output is formulated as

(2) $$X_{l}=X_{l-1}+\mathrm{GELU}\left(\mathrm{BN}\left({\mathrm{ADConv}}_{r}\left(X_{l-1}\right)\right)\right).$$

where BN denotes batch normalization, and ${\mathrm{ADConv}}_{r}\left(\cdot \right)$ denotes an asymmetric dilated convolution with dilation rate *r*. The dilation rates and repetition counts used in the three encoder stages are summarized in Table 1.

#### 3.2.3. Multi-Path Dilated Transformer

Although CADC captures local vascular structures and multi-scale spatial features, its convolutional operations remain limited in modeling long-range contextual dependencies. Therefore, an MPDT module is introduced after CADC in each encoder stage. As illustrated in Figure 3, MPDT consists of an MPDA sublayer and a feed-forward network (FFN), both implemented using pre-normalization and residual connections.

Let $X_{l}\in R^{H_{l}\times W_{l}\times C_{l}}$ denote the output of CADC, where $H_{l}$, $W_{l}$, and $C_{l}$ are the spatial dimensions and channel dimension, respectively. The normalized feature is first processed through three dilated paths. In the *r*-th path, a 3×3 convolution with dilation rate r∈{1,2,3} is applied:

(3) $$Z_{r}={\mathrm{Conv}}_{3\times 3}^{\left(r\right)}\left(\mathrm{LN}(X_{l})\right),\;r\in \left\{1,2,3\right\}.$$

The different dilation rates provide receptive fields of different spatial scales before attention computation. The resulting feature $Z_{r}$ is flattened into $N_{l}=H_{l}W_{l}$ spatial tokens and linearly projected into query, key, and value representations:

(4) $$Q_{r}=Z_{r}W_{r}^{q},\;K_{r}=Z_{r}W_{r}^{k},\;V_{r}=Z_{r}W_{r}^{v},$$

where $Q_{r},K_{r},$ and $V_{r}\in R^{N_{l}\times d_{l}}$, and $W_{r}^{q}$, $W_{r}^{k}$, and $W_{r}^{v}$ are trainable projection matrices. In the implemented MPDA, each path employs a single attention head with $d_{l}=C_{l}$. Therefore, the head dimensions are 64, 128, and 192 in Encoder Stages 1, 2, and 3, respectively.

Within each path, every query token attends to all $N_{l}=H_{l}W_{l}$ spatial tokens of the corresponding dilated feature representation. The attention output of the *r*-th path is computed as:

(5) $$A_{r}=\mathrm{Softmax}\left(\frac{Q_{r}K_{r}^{T}+B_{r}}{\sqrt{d_{l}}}\right)V_{r},$$

where $B_{r}\in R^{N_{l}\times N_{l}}$ denotes the positional encoding matrix for the *r*-th path, and $\sqrt{d_{l}}$ is the attention scaling factor.

The outputs of the three paths are concatenated along the channel dimension and projected back to $C_{l}$ channels:

(6) $$A_{\mathrm{MPDA}}=\mathrm{Concat}\left(A_{1},A_{2},A_{3}\right)W^{o},$$

where $W^{o}\in R^{3d_{l}\times C_{l}}$ is a trainable output projection matrix. The MPDA output is then combined with the CADC feature through a residual connection:

(7) $${\tilde{X}}_{l}=X_{l}+\mathrm{Reshape}\left(A_{\mathrm{MPDA}}\right).$$

Finally, a pre-normalized FFN and a second residual connection are applied:

(8) $$Y_{l}={\tilde{X}}_{l}+\mathrm{FFN}\left(\mathrm{LN}\left({\tilde{X}}_{l}\right)\right),$$

where $Y_{l}$ is the final output of the MPDT module. Thus, MPDA constitutes the attention sublayer of MPDT rather than the complete output of the MPDT block.

### 3.3. Hierarchical Context Information Transmission

In the decoder stage, simply upsampling and summing outputs from different layers often leads to suboptimal results. High-resolution predictions relying on upsampling and skip connections can introduce incorrect low-frequency information, which may degrade segmentation accuracy, especially in regions where precise boundary delineation is crucial. The hierarchical context information transmission (HCIT) module, as illustrated in Figure 2, utilizes the local variation in the low-resolution probability map to modulate the contribution of high-resolution features, emphasizing regions around predicted vessel boundaries and structural transitions.

Given four hierarchical feature maps ${\left\{H_{i}\right\}}_{i=1}^{4}$, ordered from the highest to the lowest spatial resolution, $H_{1}$ denotes the output of the convolutional stem, while $H_{2}$, $H_{3}$, and $H_{4}$ denote the outputs of Encoder Stages 1, 2, and 3, respectively. For each decoder level i∈{1,2,3}, $H_{i+1}$ denotes the corresponding lower-resolution feature map. We first apply a transposed convolution to upsample the low-resolution feature $H_{i+1}$, thereby aligning its spatial dimensions with those of the high-resolution feature $H_{i}$. The intermediate segmentation probability map $P_{i+1}$ is defined as:

(9) $$P_{i+1}=\mathrm{Seghead}\left(\mathrm{UpConv}(H_{i+1})\right).$$

Here, UpConv(·) denotes an upsampling operation implemented using a transposed convolution with trainable weights. The segmentation head processes the upsampled feature using a 1×1 convolution, a 3×3 convolution, and a sigmoid activation function to produce the probability map.

For a spatial location *x*, let Ω(x) denote its 3×3 neighborhood. The confidence mask $M_{i}$ is computed as the local range of the segmentation probability map $P_{i+1}$:

(10) $$\begin{array}{cc} M_{i}\left(x\right) & =\underset{u\in \Omega (x)}{\mathrm{MaxPool}}\left(P_{i+1}\left(u\right)\right)-\underset{u\in \Omega (x)}{\mathrm{MinPool}}\left(P_{i+1}\left(u\right)\right)\\ \; & =\underset{u\in \Omega (x)}{\mathrm{MaxPool}}\left(P_{i+1}\left(u\right)\right)+\underset{u\in \Omega (x)}{\mathrm{MaxPool}}\left(-P_{i+1}\left(u\right)\right). \end{array}$$

where MaxPool(·) and MinPool(·) denote the maximum and minimum operations, respectively, over the local neighborhood Ω(x). The subtraction “−” and addition “+” operations above are performed element-wise on the probability map. A larger value of $M_{i}\left(x\right)$ indicates stronger local variation between vessel-like and background-like responses, which commonly occurs around vessel boundaries or structural transitions. Conversely, a smaller value indicates a more spatially uniform probability response. Therefore, $M_{i}$ represents the local variation of the predicted probabilities.

The confidence mask $M_{i}$ is then used to modulate the high-resolution feature $H_{i}$. Specifically, a 1×1 convolution is first applied to $H_{i}$, after which the mask is broadcast along the channel dimension and multiplied element-wise with the transformed feature:

(11) $$M_{i}^{\prime }=M_{i}⊙{\mathrm{Conv}}_{1\times 1}\left(H_{i}\right),$$

where ⊙ denotes element-wise multiplication, and $M_{i}^{\prime }$ denotes the mask-modulated high-resolution feature.

To enhance cross-layer information flow, $M_{i}^{\prime }$ is subsequently fused with the upsampled low-resolution feature:

(12) $$M_{i}^{\prime \prime }=\mathrm{UpConv}\left(H_{i+1}\right)⊕M_{i}^{\prime },$$

where $M_{i}^{\prime \prime }$ denotes the fused output feature of the HCIT module. ⊕ denotes element-wise addition of feature tensors. This operation uses the local probability variation encoded by $M_{i}$ to modulate the introduction of high-resolution information, thereby facilitating hierarchical feature integration in regions containing vessel boundaries and structural transitions.

### 3.4. Loss Function

In the context of medical image segmentation tasks, two commonly employed loss functions are the cross-entropy loss $L_{ce}$ and Dice loss $L_{dice}$ [27]. These functions are instrumental in optimizing the performance of segmentation models by measuring the discrepancy between predicted and ground truth. The corresponding definitions are:

(13) $$\begin{array}{cc} L_{ce} & =-\frac{1}{N}\sum_{i=1}^{N}\left(y_{i}\log{\hat{y}}_{i}+\left(1-y_{i}\right)\log\left(1-{\hat{y}}_{i}\right)\right) \end{array}$$

(14) $$\begin{array}{cc} L_{dice} & =1-\frac{2\sum_{i=1}^{N}y_{i}{\hat{y}}_{i}}{\sum_{i=1}^{N}y_{i}+\sum_{i=1}^{N}{\hat{y}}_{i}} \end{array}$$

where ${\hat{y}}_{i}$ and $y_{i}$ denote the predicted and true labels of the *i*-th pixel, respectively, and *N* represents the total number of pixels. $L_{ce}$ evaluates the performance of a classification model whose output is a probability value between 0 and 1. It penalizes the predictions based on the deviation from the actual labels. $L_{dice}$ directly measures the overlap between the predicted segmentation and the ground truth. It ranges from 0 to 1, with a dice loss of 0 indicating perfect overlap.

The objective loss function $L_{total}$ is defined as the sum of the commonly used cross-entropy loss and soft dice loss to leverage the benefits of both complementary loss functions simultaneously. It can be defined as

(15) $$L_{total}=L_{ce}+L_{dice}$$

The pixel-wise classification accuracy of the cross-entropy loss and the spatial overlap optimization of the dice loss provide a balanced approach, thus guiding the model to minimize the prediction error over the training dataset.

## 4. Experiments

### 4.1. Datasets

**Figure 4:**
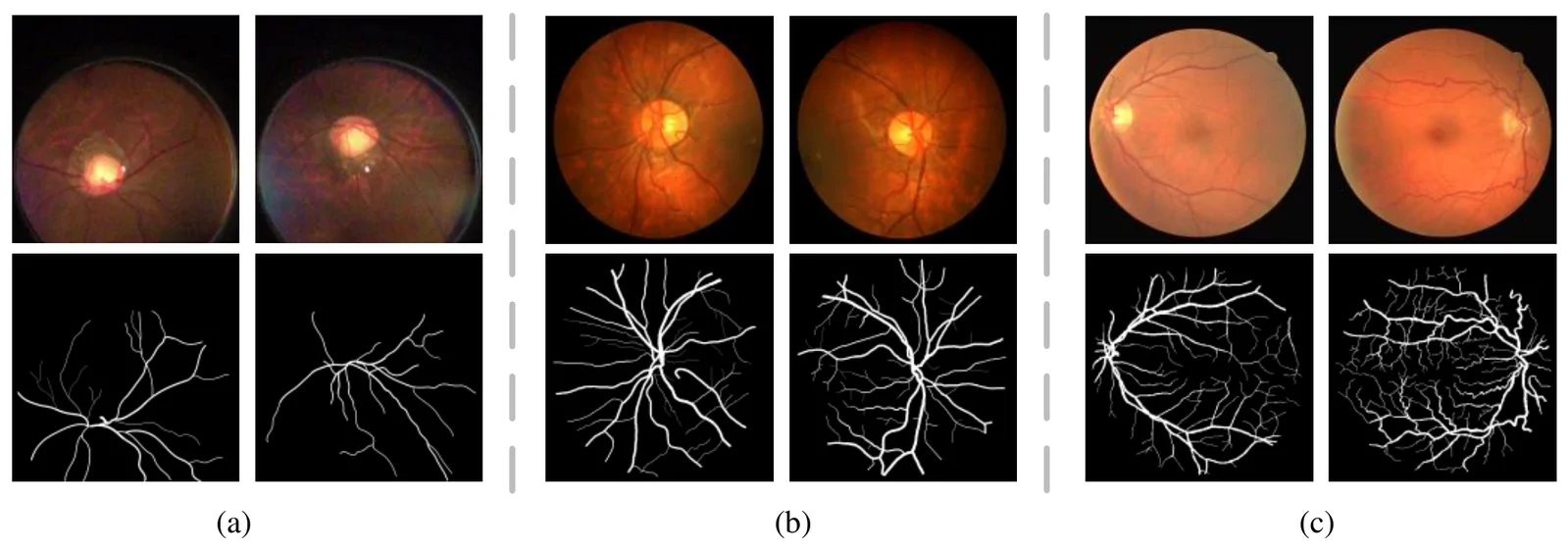
Cases of vessel segmentation. (**a**) RVD, (**b**) CHASE_DB1, (**c**) DRIVE. The original images are on the top, and the corresponding ground-truth images are on the bottom.

In this study, we utilize the RVD dataset [44] captured by smartphone-based devices. Specifically, it is implemented by attaching a smartphone to a fundus camera lens, and trained clinicians operate the device to capture fundus images of patients. In addition, two widely used datasets—CHASE_DB1 [45] and DRIVE [46], acquired from bench-top devices—are included to broaden the scope of the analysis, thereby validating the reliability and applicability of the proposed method. The specifications of these datasets are summarized in Table 2, and some typical images are given in Figure 4.

The public RVD dataset [44] is the first video-based retinal dataset captured by smartphone-based handheld devices. It comprises 635 videos collected from 415 patients across four different clinics. To mitigate the redundancy of annotating similar video frames, two keyframes are selected from each video for spatial annotation, resulting in a total of 1270 images with a resolution of 1800×1800 pixels. Following the official protocol, the dataset is split at the patient ID level to ensure that videos from the same subject do not simultaneously appear in both training and test sets, effectively preventing data leakage. Specifically, the official split allocates 970 images for training, 64 for validation, and 236 for testing.

The DRIVE dataset came from a screening program for diabetic retinopathy in the Netherlands. It includes 40 color images, 20 for training and 20 for testing. The CHASE_DB1 dataset is captured with a $30^{\circ }$ FOV centered at the optic disc, including 28 retinal images with a resolution of 999×960 pixels. For our experiments, the 20 development images were divided into 18 training images and 2 validation images, while the remaining 8 images were reserved exclusively for testing.

### 4.2. Evaluation Metrics

Retinal vessel segmentation is formulated as a pixel-wise binary classification task, in which each pixel is assigned to either the vessel class or the background class. Based on the predicted and ground-truth labels, the segmentation outcomes are categorized as true positives (TP), true negatives (TN), false positives (FP), and false negatives (FN). Specifically, TP and TN denote correctly identified vessel and background pixels, respectively, whereas FP denotes background pixels misclassified as vessels, and FN denotes vessel pixels misclassified as background. Specifically, for each independent run, we accumulated TP, TN, FP, and FN across all evaluated pixels in the entire test set. Accuracy (ACC), sensitivity (SE), specificity (SP), and the vessel-class F1-score (F1) were then calculated from this pooled confusion matrix using a fixed binarization threshold of 0.5 on the predicted probability maps. AUC was calculated from the continuous pixel-wise prediction probabilities before thresholding by constructing the ROC curve over all evaluated pixels in the test set. To assess whether the performance differences were statistically significant, two-sided paired *t*-tests were conducted using the results from five matched random seeds, namely {0,1,2,3,4}. For each metric, the result obtained by HiRNet under a given seed was paired with the result obtained by the strongest competing method under the same seed.

The results are reported as mean ± standard deviation across the five runs.

(16) $$\begin{array}{cc} ACC & =\frac{TP+TN}{TP+TN+FP+FN}, \end{array}$$

(17) $$\begin{array}{cc} SE & =\frac{TP}{TP+FN}, \end{array}$$

(18) $$\begin{array}{cc} SP & =\frac{TN}{TN+FP}, \end{array}$$

(19) $$\begin{array}{cc} F1 & =\frac{2TP}{2TP+FP+FN}, \end{array}$$

(20) $$\begin{array}{cc} AUC & =\frac{\sum_{i=1}^{M}rank_{i}-\frac{M(M+1)}{2}}{MN}. \end{array}$$

where *M* and *N* denote the numbers of vessel and background pixels, and $rank_{i}$ denotes the rank assigned to the predicted probability of the *i*-th vessel pixel among the prediction probabilities of all M+N evaluated pixels. For the formulation in Equation (20), the continuous prediction probabilities are ranked in ascending order, with the lowest probability assigned rank 1. Equal prediction probabilities are assigned their average rank.

To further assess threshold-independent performance, average precision (AP) was also calculated from the precision–recall curve using the continuous pixel-wise prediction probabilities and binary ground-truth labels. For each independent run, the prediction probabilities and labels were pooled over all pixels in the test set before calculating AP. The final AP values are reported as the mean ± standard deviation over five independent runs.

### 4.3. Implementation Details

We trained the HiRNet model on NVIDIA 4090 graphics with 24 GB memory and 128 GB of RAM. For our implementation, Python 3.9 and PyTorch 2.0 were employed. All images in the RVD dataset are resized to 512×512 pixels for model training, while the CHASE_DB1 and DRIVE datasets employ patch-based training using 128×128 patches extracted via sliding window cropping with step sizes of 16 and 8 pixels, respectively. Importantly, the training/validation split was performed at the complete-image level before patch extraction. Sliding-window cropping was then applied independently to the training and validation images. Consequently, all overlapping patches extracted from a given retinal image belonged exclusively to one subset, and no patches from the same image appeared in both the training and validation sets. The test images were not involved in either model training or validation. The data augmentations such as horizontal flip, vertical flip, rotation, and cutout with the probability of 0.25 are used to increase the data diversity. Prior to training, the epoch was set to 150, the Adam optimizer was employed, and the learning rate was initialized to $2\times 10^{-5}$ with an exponential decay rate of 0.9. During training, we reserved 10% of the DRIVE and CHASE_DB1 datasets for validation purposes, while the RVD dataset maintained its original predefined train/validation/test split. We implemented early stopping based on the F-measure validation performance, terminating training when the metric failed to improve for 5 consecutive epochs.

For a fair comparison, all methods were reimplemented and trained using the same dataset splits, preprocessing procedures, image resolutions, data augmentation strategies, and early-stopping criterion as HiRNet. For methods with publicly available implementations, we followed their official implementations and retained the recommended architecture-specific hyperparameters whenever applicable. All experiments were independently repeated five times using different random seeds, and the mean and standard deviation are reported. The random seeds were fixed to 0, 1, 2, 3, 4 for model initialization, data shuffling, and augmentation sampling.

### 4.4. Comparisons with State-of-the-Art Methods

**Figure 5:**
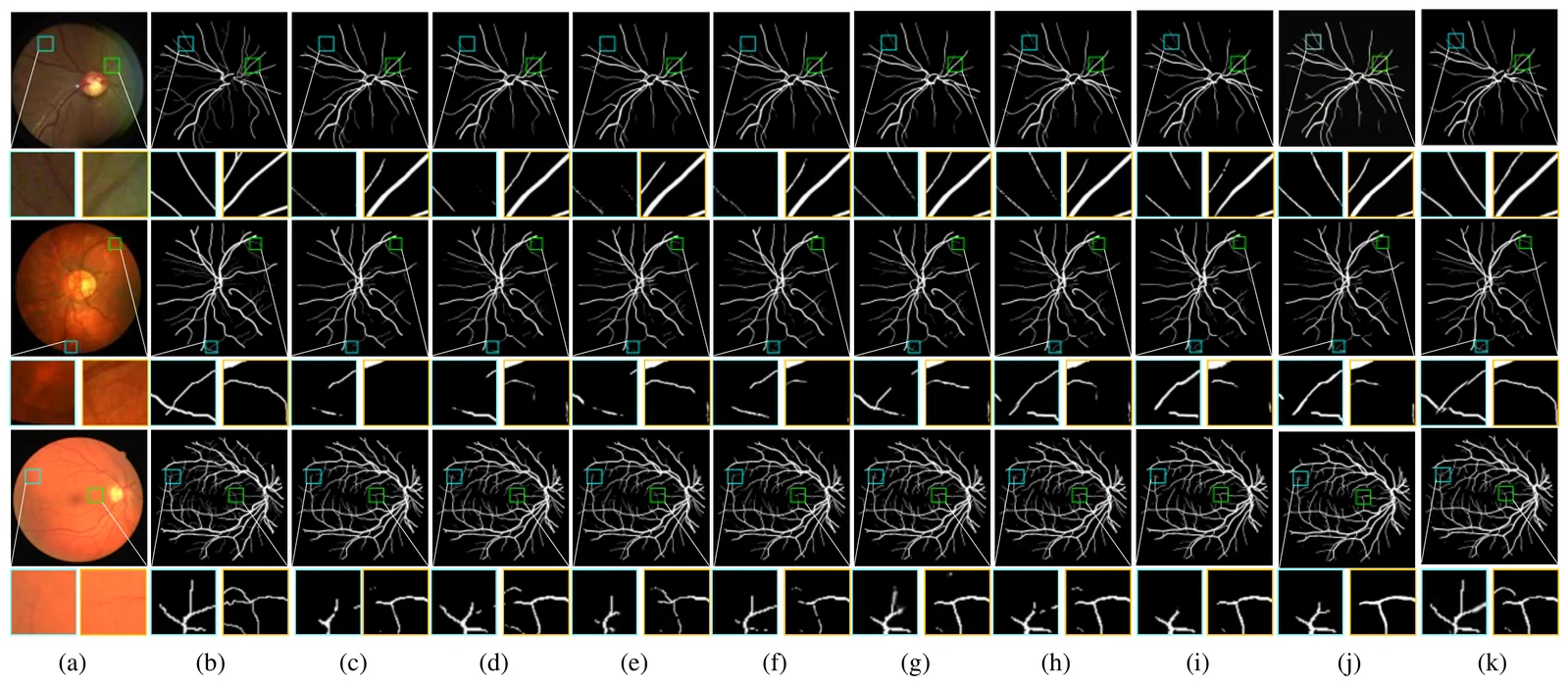
Visualization results of different methods on retinal vessel datasets. (**a**) Original images, (**b**) ground truth, (**c**) U-Net, (**d**) UNet++, (**e**) AG-Net, (**f**) DeepLabV3+, (**g**) SegFormer, (**h**) Mask2Former, (**i**) MGA-Net, (**j**) IMFF-Net, and (**k**) HiRNet (ours).

We conducted a comprehensive comparative analysis with general medical image segmentation methods, i.e., U-Net [13], DeepLabV3+ [47], SegFormer [48] and Mask2Former [49], recommended by RVD. For specialized retinal vessel segmentation approaches, we compared our model against UNet++ [18], AG-Net [9], MGA-Net [50] and IMFF-Net [41]. Table 3, Table 4 and Table 5 present the quantitative results on the RVD, DRIVE, and CHASE_DB1 datasets, respectively. On the RVD dataset, HiRNet obtained the highest mean values for SE, SP, ACC, F1-score, AUC, and AP, reaching 58.36%, 97.81%, 95.83%, 58.39%, 95.16%, and 64.87%, respectively. The improvements in SE, ACC, F1-score, AUC, and AP were statistically significant, whereas the improvement in SP was not statistically significant. On the DRIVE dataset, HiRNet obtained the highest mean SE, ACC, F1-score, AUC, and AP values of 82.73%, 95.59%, 82.76%, 98.83%, and 87.94%, respectively. The improvements in SE, ACC, F1-score, and AP were statistically significant, whereas the differences in SP and AUC were not statistically significant. On the CHASE_DB1 dataset, HiRNet obtained the highest mean SE, ACC, F1-score, AUC, and AP values of 82.13%, 95.98%, 80.37%, 97.90%, and 86.82%, respectively. The improvements in SE, F1-score, and AUC were statistically significant, whereas the differences in SP, ACC, and AP were not statistically significant. This indicates that using the hybrid CNN–Transformer feature and hierarchical refinement as core building blocks to design the U-shaped encoder-decoder structure may be helpful for learning hierarchical information. It can be seen that U-shaped methods, i.e., U-Net, U-Net++, AG-Net, MGA-Net and IMFF-Net, do not show advantages across all datasets. The main reason is that skip connections retain spatial information by merging high-resolution features from the encoder with low-resolution features from the decoder, but they do not inherently address global context. Also, the U-shaped models leverage encoder–decoder structures with skip connections to mitigate multi-scale feature loss caused by downsampling, the convolution operations sometimes struggle to capture global contextual information. Although the guided filter in AG-Net helps to focus on important regions, the comprehensive capture of global contextual information is still weak. In most cases, DeepLabV3+ shows competitive performance on some metrics, but its overall performance remains lower than that of the proposed method. This structure leverages multiple scales of atrous convolutions to capture features at different levels, allowing the network to handle complex scenes and details more effectively. Regarding MGA-Net and IMFF-Net, while specifically designed for vessel segmentation, these architectures fail to adequately account for the unique characteristics of smartphone-captured images, particularly their lower quality and different noise profiles compared to standard imaging modalities. In contrast, the superior performance of the proposed method stems from multi-scale feature extraction and multi-path dilated attention structure. Additionally, the proposed method refines segmentation progressively at multiple levels, enabling precise delineation of vessel boundaries. This multi-level refinement approach helps in addressing both coarse and fine segmentation challenges, resulting in more accurate and reliable retinal vessel segmentation. We also noted that HiRNet does not achieve the highest SP on DRIVE or CHASE_DB1. The slightly lower SP indicates that a modestly larger proportion of background pixels is classified as vessels. Additionally, the inherent trade-off between sensitivity and specificity [51,52] suggests that prioritizing higher sensitivity may lead to reduced specificity, potentially explaining the slightly lower specificity. Nevertheless, while specificity is an important metric, the overall strong performance in other metrics, such as F1 and AUC, indicates that the model maintains a well-balanced approach. Moreover, we further visualize results in Figure 5, where it can be observed that the proposed HiRNet delivers more complete vascular structures and improves vessel continuity.

To evaluate the computational complexity of the proposed method, we compare the number of trainable parameters, FLOPs, and indicative inference time of HiRNet with those of the competing methods. As shown in Table 6, HiRNet requires 18.92 M parameters and 122.96 G FLOPs, representing the lowest parameter count and computational cost among the evaluated methods. Compared with SegFormer, which has the second-lowest parameter count, HiRNet reduces the number of parameters by 20.24%. In terms of FLOPs, HiRNet requires 10.56% fewer computations than DeepLabV3+, which has the second-lowest computational cost. Compared with U-Net, HiRNet reduces the parameter count and FLOPs by 39.05% and 43.86%, respectively. The inference times are retained as indicative reference values and are not used to support claims regarding statistically validated differences in inference speed.

### 4.5. Ablation Study on Model Architectures

**Figure 6:**
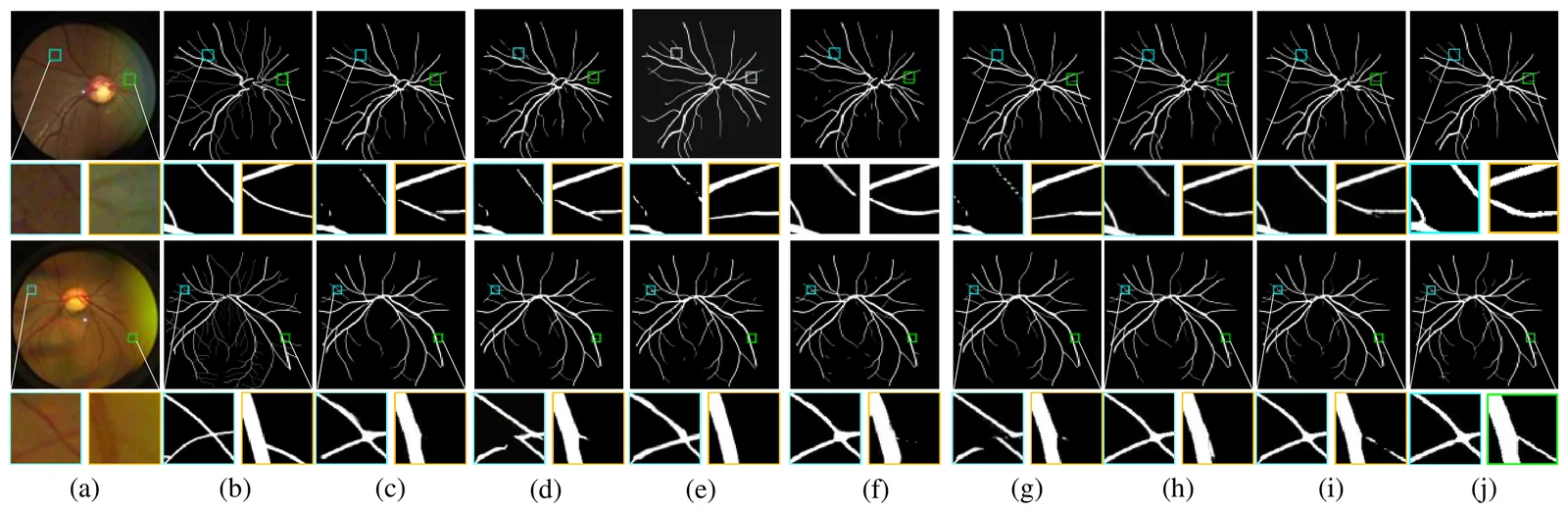
Qualitative results of selected cumulative and leave-one-out ablation configurations on the RVD dataset: (**a**) Original images, (**b**) ground truth, (**c**) Baseline, (**d**) Baseline + BD, (**e**) Baseline + BD + CADC, (**f**) Baseline + BD + CADC + MPDT, (**g**) Full w/o BD, (**h**) Full w/o CADC, (**i**) Full w/o MPDT, and (**j**) Baseline + BD + CADC + MPDT + HCIT (Full).

To investigate the contributions of the proposed architectural components, we conducted cumulative and leave-one-out ablation studies on the RVD dataset. The quantitative and qualitative results are presented in Table 7 and Figure 6, respectively. The baseline retains the same convolutional stem, three-stage encoder–decoder topology, channel dimensions, and stage depths as the complete HiRNet, but excludes BD, CADC, MPDT, and HCIT. The three encoder stages use 64, 128, and 192 channels and contain 3, 3, and 6 blocks, respectively. Spatial downsampling is performed using a standard 3×3 convolution with stride 2, without Gaussian filtering or average pooling. Each encoder stage consists of standard residual convolutional blocks [53] composed of 3×3 convolutions, batch normalization, GELU activation, and an identity shortcut. No asymmetric dilated convolution or self-attention is used in the baseline.

Before HCIT is introduced, the baseline employs a conventional decoder. At each decoder level, a stride-2 transposed convolution upsamples the lower-resolution feature. The upsampled feature is combined with the corresponding encoder feature through element-wise addition and then processed by a standard residual convolutional block. Finally, a segmentation head maps the decoder feature to a one-channel probability map.

As shown in Table 7, the cumulative configurations describe the progressive construction of HiRNet. Compared with the baseline, adding BD increases the mean SE, SP, ACC, F1-score, and AUC by 1.15, 0.25, 0.29, 1.89, and 0.28 percentage points, respectively. Adding CADC to the BD configuration further increases these metrics by 0.40, 0.93, 0.91, 4.79, and 0.28 percentage points, respectively. These results suggest that blur-based downsampling and asymmetric dilated convolutions provide complementary benefits for preserving spatial information and modeling multi-scale vascular structures.

Adding MPDT to the Baseline + BD + CADC configuration increases the mean SE and AUC by 1.66 and 0.45 percentage points, respectively. However, the mean SP, ACC, and F1-score decrease by 0.38, 0.28, and 0.77 percentage points, respectively. This result indicates a trade-off between vessel sensitivity and the control of false-positive predictions at the fixed threshold of 0.5. The increase in AUC suggests that MPDT improves vessel–background ranking performance across different decision thresholds, although this improvement does not lead to uniform gains in threshold-dependent metrics.

Adding HCIT to the Baseline + BD + CADC + MPDT configuration increases the mean SE, SP, ACC, F1-score, and AUC by 0.84, 0.47, 0.48, 3.11, and 0.38 percentage points, respectively. The complete HiRNet achieves the highest observed mean values for all five evaluation metrics, reaching 58.36% in SE, 97.81% in SP, 95.83% in ACC, 58.39% in F1-score, and 95.16% in AUC.

The leave-one-out configurations assess the effect of removing one component from the complete HiRNet while retaining the other components. Relative to the complete model, removing BD decreases the mean SE, SP, ACC, F1-score, and AUC by 2.01, 0.56, 0.53, 5.49, and 0.71 percentage points, respectively. Removing CADC decreases these metrics by 2.68, 0.96, 1.03, 6.84, and 1.04 percentage points, respectively. Removing MPDT decreases them by 2.54, 0.89, 0.95, 6.61, and 0.96 percentage points, respectively. The configuration without HCIT is represented by the cumulative configuration Baseline + BD + CADC + MPDT. Compared with this configuration, the complete HiRNet increases the mean SE, SP, ACC, F1-score, and AUC by 0.84, 0.47, 0.48, 3.11, and 0.38 percentage points, respectively. These results show that removing any of the evaluated components is associated with lower mean performance under the tested configurations.

Notably, Full w/o MPDT performs below Baseline + BD + CADC on all five metrics, although the former retains HCIT. This comparison indicates that the benefit of HCIT is context-dependent and, in the evaluated architecture, relies on the contextual representation supplied by MPDT rather than constituting an independent gain.

The cumulative comparisons reflect the conditional effect of adding a component after the preceding components have already been introduced. Similarly, the leave-one-out results reflect the effect of removing a component within the specific context of the complete HiRNet. Therefore, these results should not be interpreted as universal, context-independent causal estimates. In particular, the current experiments do not fully quantify interactions among components or the dependence of their effects on the order of introduction. A complete factorial analysis would be required to estimate these interaction and order effects separately.

### 4.6. Cross-Dataset Validation

In addition, we conducted cross-dataset experiments between the smartphone-based RVD dataset and two conventional fundus datasets, DRIVE and CHASE_DB1. Four transfer settings were considered: DRIVE → RVD, CHASE_DB1 → RVD, RVD → DRIVE, and RVD → CHASE_DB1. In each setting, the model was trained and validated exclusively on the source dataset and directly evaluated on the target test set without fine-tuning or domain adaptation. No target-domain images or annotations were used during training, validation, hyperparameter optimization, or model selection.

As shown in Table 8, HiRNet achieved the highest F1-score and AUC in all four transfer settings. When transferring from the conventional fundus datasets to the smartphone-based RVD dataset, HiRNet obtained an F1-score of 47.61% and an AUC of 91.36% under the DRIVE → RVD setting. Compared with the strongest competing method, Mask2Former, these results represent improvements of 4.41 percentage points in F1-score and 1.53 percentage points in AUC. Under the CHASE_DB1 → RVD setting, HiRNet achieved an F1-score of 49.84% and an AUC of 92.08%, exceeding Mask2Former by 4.96 and 1.67 percentage points, respectively. HiRNet also demonstrated favorable performance in the reverse transfer directions. Under the RVD → DRIVE setting, it achieved an F1-score of 73.88% and an AUC of 96.10%, outperforming Mask2Former by 3.58 and 1.48 percentage points, respectively. Similarly, under the RVD → CHASE_DB1 setting, HiRNet obtained an F1-score of 69.85% and an AUC of 95.20%, corresponding to improvements of 3.85 percentage points in F1-score and 1.44 percentage points in AUC over Mask2Former. HiRNet also exhibited relatively small standard deviations across the five independent runs, indicating stable performance under the evaluated cross-dataset settings.

The lower F1-scores observed when transferring from DRIVE or CHASE_DB1 to RVD reflect the greater domain shift associated with smartphone-based fundus images, including differences in illumination, contrast, field of view, and imaging artifacts. In contrast, the relatively high AUC values indicate that the models retain useful vessel–background discrimination across domains, although selecting a fixed decision threshold remains more challenging on RVD. The consistent performance of HiRNet in both transfer directions suggests that its integration of multi-scale local features, global contextual information, and hierarchical refinement improves robustness to variations between smartphone-based and conventional fundus imaging.

## 5. Discussion

Although the proposed HiRNet demonstrates strong overall performance on three publicly available retinal vessel datasets, several aspects of its results warrant further discussion. As shown in Table 3, Table 4 and Table 5, HiRNet achieves the highest mean SE, SP, ACC, F1, and AUC on RVD. On DRIVE and CHASE_DB1, it achieves the highest mean SE, ACC, F1, and AUC, although its SP is slightly lower than the best competing result. Specifically, the SP of HiRNet is 0.18 percentage points lower than that of MGA-Net on DRIVE and 0.04 percentage points lower than that of DeepLabV3+ on CHASE_DB1. This result reflects the inherent trade-off between sensitivity and specificity at a fixed decision threshold. The higher sensitivity achieved by HiRNet may be accompanied by a modest increase in false-positive vessel predictions, leading to a slightly lower SP. Nevertheless, its strong ACC, F1, and AUC indicate a favorable overall balance between vessel detection and background suppression.

The observed improvements are associated with the complementary integration of multi-scale local feature extraction, global contextual modeling, and hierarchical feature refinement. Specifically, CADC captures vascular structures at different receptive-field scales, whereas MPDT models broader contextual relationships through multiple attention paths. HCIT further integrates features from different resolutions by using the local variation in the low-resolution probability map to modulate high-resolution features. The resulting mask should therefore be interpreted as a local probability-variation measure rather than conventional prediction confidence. It produces stronger responses around predicted vessel boundaries and structural transitions while suppressing high-resolution responses in locally homogeneous regions. The ablation results in Table 7 further support the complementary roles of the proposed components. Although MPDT introduces modest decreases in SP, ACC, and F1 at the fixed threshold, it improves SE and AUC. In the tested cumulative configuration, adding HCIT after MPDT improves all five metrics. However, the lower performance of Full w/o MPDT than Baseline + BD + CADC indicates that this benefit depends on the presence of MPDT. Compared with the baseline, the complete HiRNet improves SE, SP, ACC, F1, and AUC by 4.05, 1.27, 1.40, 9.02, and 1.39 percentage points, respectively.

The cross-dataset experiments in Table 8 provide additional evidence of the robustness of HiRNet to domain shifts between smartphone-based and conventional fundus images. HiRNet achieves the highest mean F1 and AUC in all four evaluated transfer settings without target-domain fine-tuning or domain adaptation. Nevertheless, RVD is the only smartphone-based fundus dataset included in this study, and DRIVE and CHASE_DB1 contain relatively few images. Therefore, the present results do not fully establish robustness across different smartphone models, optical adapters, clinical centers, or patient populations. Future studies should evaluate HiRNet on larger multicenter datasets acquired using different smartphone devices and under diverse imaging conditions. Cross-device validation, domain adaptation, prediction calibration, and uncertainty estimation may further improve its reliability in practical applications. Although HiRNet has a relatively compact architecture and low theoretical computational complexity, lightweight network design, model compression, and direct evaluation on portable hardware remain necessary before deployment in smartphone-based fundus screening systems.

Despite the relative improvement over the evaluated competing methods, the sensitivity and F1 values of approximately 58% on RVD are not adequate for unsupervised or autonomous clinical use. The present contribution should therefore be interpreted as an algorithmic improvement on a difficult smartphone-acquired imaging domain rather than evidence of clinical readiness. Prospective multicenter and cross-device validation, together with calibration, uncertainty analysis, and evaluation in clinician-supervised workflows, is required before practical deployment.

## 6. Conclusions

In this paper, we propose HiRNet, a hierarchical-refinement-guided hybrid CNN–Transformer network for retinal vessel segmentation in smartphone-based fundus images. HiRNet combines a blur downsampler, consecutive asymmetric dilated convolutions, a multi-path dilated Transformer, and hierarchical context information transmission. The blur downsampler is used to alleviate aliasing artifacts during downsampling, while CADC captures multi-scale local vascular representations. MPDT models broader contextual relationships through multiple attention paths. However, the incremental ablation shows that MPDT does not uniformly improve all evaluation metrics: it increases SE and AUC but slightly decreases SP, ACC, and F1 at the fixed threshold of 0.5. This result indicates a trade-off between detecting additional vessel pixels and controlling false-positive predictions. In the tested cumulative configuration, adding HCIT after MPDT improves all five metrics. However, the lower performance of Full w/o MPDT than Baseline + BD + CADC indicates that this benefit depends on the presence of MPDT. Experiments on RVD, DRIVE, and CHASE_DB1 demonstrate that the complete HiRNet achieves strong overall segmentation performance. Nevertheless, the sensitivity and F1 values of approximately 58% on RVD are not sufficient for unsupervised clinical deployment. The results should be interpreted as a relative improvement on a challenging smartphone-acquired imaging domain. Further prospective, multicenter, cross-device, hardware-specific, and clinician-in-the-loop validation is required before clinical use.

## Figures and Tables

**Table 1:** Detailed configuration of the HiRNet encoder. The notation [(3×1,C)→(1×3,C)]×T denotes *T* consecutively connected ADConv blocks. Each block consists of a vertical 3×1 convolution followed by a horizontal 1×3 convolution and produces *C* output channels. Thus, the asymmetric kernel length is k=3. Each encoder stage contains a CADC module and an MPDT module as two separate components. The output of CADC is subsequently fed into MPDT. The dilation rates listed for CADC correspond to the successive ADConv blocks, whereas those listed for MPDA correspond to its three parallel paths. MPDA serves as the core attention mechanism of MPDT.

Layers	HiRNet Configuration
Conv Stem	3×3, 64, stride =2
	[3×3,64]×2
Blur Downsampling 1	3×3, 64; ${\mathrm{Blur}}_{4\times 4}$; AvgPool
Stage 1 CADC	[(3×1,64)→(1×3,64)]×3, dilation =(1,2,3)
Stage 1 MPDT	MPDA with three parallel 3×3
	dilated-convolution paths, dilation =(1,2,3)
Blur Downsampling 2	3×3, 128; ${\mathrm{Blur}}_{4\times 4}$; AvgPool
Stage 2 CADC	[(3×1,128)→(1×3,128)]×3, dilation =(1,2,3)
Stage 2 MPDT	MPDA with three parallel 3×3
	dilated-convolution paths, dilation =(1,2,3)
Blur Downsampling 3	3×3, 192; ${\mathrm{Blur}}_{4\times 4}$; AvgPool
Stage 3 CADC	[(3×1,192)→(1×3,192)]×6, dilation =(1,2,3,2,4,6)
Stage 3 MPDT	MPDA with three parallel 3×3
	dilated-convolution paths, dilation =(1,2,3)

**Table 2:** Summary of the retinal vessel datasets.

Dataset	Total	Training	Validation	Testing
RVD	1270	970	64	236
DRIVE	40	18	2	20
CHASE_DB1	28	18	2	8

**Table 3:** Segmentation performance on the RVD dataset. Boldface and underlining indicate the highest and second-highest mean values in each column, respectively. Statistical comparisons were conducted between HiRNet and the underlined results using two-sided paired *t*-tests across five matched random seeds. * indicates a statistically significant improvement (p<0.05), whereas ^†^ indicates a non-significant difference (p≥0.05).

Model	SE (%)	SP (%)	Acc (%)	F1 (%)	AUC (%)	AP (%)
U-Net [13]	57.31 ± 1.79	95.54 ± 1.24	93.63 ± 1.12	47.36 ± 1.31	93.81 ± 0.48	54.21 ± 1.28
UNet++ [18]	56.87 ± 1.53	96.81 ± 0.64	94.80 ± 0.58	52.29 ± 1.18	93.74 ± 0.52	58.93 ± 1.15
AG-Net [9]	56.69 ± 1.41	96.76 ± 0.58	94.77 ± 0.49	51.95 ± 1.10	92.89 ± 0.61	58.27 ± 1.08
DeepLabV3+ [47]	57.39 ± 1.27	97.47 ± 0.42	95.46 ± 0.36	55.87 ± 0.96	94.35 ± 0.39	62.34 ± 0.93
SegFormer [48]	54.29 ± 1.16	96.79 ± 0.55	94.66 ± 0.42	50.44 ± 0.91	92.54 ± 0.46	56.98 ± 0.87
Mask2Former [49]	53.86 ± 1.09	96.77 ± 0.31	94.62 ± 0.28	50.05 ± 0.82	93.22 ± 0.43	56.72 ± 0.79
MGA-Net [50]	56.36 ± 1.68	96.79 ± 1.37	94.78 ± 1.23	51.86 ± 1.42	94.32 ± 0.77	58.16 ± 1.36
IMFF-Net [41]	56.18 ± 1.72	96.29 ± 0.83	94.29 ± 0.71	49.57 ± 1.36	94.16 ± 0.94	56.43 ± 1.31
Ours	**58.36 ± 0.92** *	**97.81 ± 0.28** ^†^	**95.83 ± 0.22** *	**58.39 ± 0.74** *	**95.16 ± 0.27** *	**64.87 ± 0.71** *

**Table 4:** Segmentation performance on the DRIVE dataset. Boldface and underlining indicate the highest and second-highest mean values in each column, respectively. Statistical comparisons were conducted between HiRNet and the underlined results using two-sided paired *t*-tests across five matched random seeds. For SP, HiRNet was compared with MGA-Net. * indicates a statistically significant difference (p<0.05), whereas ^†^ indicates a non-significant difference (p≥0.05).

Model	SE (%)	SP (%)	Acc (%)	F1 (%)	AUC (%)	AP (%)
U-Net [13]	77.96 ± 1.24	97.25 ± 0.27	94.78 ± 0.31	79.27 ± 0.86	97.56 ± 0.32	84.52 ± 0.79
UNet++ [18]	77.18 ± 1.12	97.44 ± 0.31	94.85 ± 0.28	79.32 ± 0.79	98.29 ± 0.26	84.89 ± 0.72
AG-Net [9]	79.29 ± 0.96	97.16 ± 0.46	94.85 ± 0.27	79.89 ± 0.72	98.49 ± 0.41	85.37 ± 0.65
DeepLabV3+ [47]	80.22 ± 1.03	97.28 ± 0.52	95.09 ± 0.30	80.74 ± 0.77	97.02 ± 0.37	86.12 ± 0.71
SegFormer [48]	79.87 ± 0.91	97.41 ± 0.39	95.16 ± 0.23	80.86 ± 0.68	97.07 ± 0.24	86.24 ± 0.63
Mask2Former [49]	80.55 ± 0.88	97.33 ± 0.25	95.18 ± 0.20	81.05 ± 0.65	97.28 ± 0.33	86.41 ± 0.59
MGA-Net [50]	80.32 ± 0.82	**97.66 ± 0.41**	95.43 ± 0.24	81.84 ± 0.64	98.18 ± 0.29	87.02 ± 0.56
IMFF-Net [41]	80.74 ± 0.76	97.51 ± 0.30	95.38 ± 0.19	81.77 ± 0.59	98.42 ± 0.25	86.98 ± 0.53
Ours	**82.73 ± 0.69** *	97.48 ± 0.24 ^†^	**95.59 ± 0.17** *	**82.76 ± 0.58** *	**98.83 ± 0.18** ^†^	**87.94 ± 0.49** *

**Table 5:** Segmentation performance on the CHASE_DB1 dataset. Boldface and underlining indicate the highest and second-highest mean values in each column, respectively. Statistical comparisons were conducted between HiRNet and the underlined results using two-sided paired *t*-tests across five matched random seeds. For SP, HiRNet was compared with DeepLabV3+. * indicates a statistically significant difference (p<0.05), whereas ^†^ indicates a non-significant difference (p≥0.05).

Model	SE (%)	SP (%)	Acc (%)	F1 (%)	AUC (%)	AP (%)
U-Net [13]	80.65 ± 1.21	96.92 ± 0.38	95.29 ± 0.26	77.41 ± 0.83	97.55 ± 0.27	83.86 ± 0.76
UNet++ [18]	80.97 ± 1.08	96.26 ± 0.31	94.73 ± 0.22	75.46 ± 0.74	97.72 ± 0.25	82.13 ± 0.68
AG-Net [9]	80.86 ± 0.94	96.55 ± 0.27	95.01 ± 0.20	76.38 ± 0.68	97.27 ± 0.29	82.84 ± 0.62
DeepLabV3+ [47]	81.11 ± 1.03	**97.58 ± 0.61**	95.91 ± 0.35	79.97 ± 0.88	97.04 ± 0.36	86.24 ± 0.82
SegFormer [48]	80.13 ± 0.91	97.12 ± 0.54	95.45 ± 0.31	77.72 ± 0.81	97.22 ± 0.31	84.28 ± 0.74
Mask2Former [49]	81.19 ± 0.86	97.32 ± 0.33	95.71 ± 0.23	79.11 ± 0.70	97.28 ± 0.34	85.57 ± 0.65
MGA-Net [50]	81.31 ± 0.89	97.33 ± 0.29	95.75 ± 0.22	79.32 ± 0.66	97.41 ± 0.28	85.74 ± 0.61
IMFF-Net [41]	81.74 ± 1.02	97.57 ± 0.26	95.94 ± 0.21	80.25 ± 0.76	97.75 ± 0.24	86.57 ± 0.70
Ours	**82.13 ± 0.72** *	97.54 ± 0.21 ^†^	**95.98 ± 0.16** ^†^	**80.37 ± 0.57** *	**97.90 ± 0.18** *	**86.82 ± 0.53** ^†^

**Table 6:** Computational complexity and indicative inference-time comparison. Parameters and FLOPs are calculated using an input resolution of 512×512, where one multiply–accumulate operation is counted as two floating-point operations. Inference times were measured on an NVIDIA RTX 4090 GPU with a batch size of 1. Because no standardized warm-up protocol or repeated timing trials were used, the reported times are only indicative and are not intended for statistical comparisons of inference speed.

Methods	Params (M)	FLOPs (G)	Time (ms)
U-Net [13]	31.04	219.04	51.83
UNet++ [18]	36.63	285.34	56.42
AG-Net [9]	34.89	242.70	54.16
DeepLabV3+ [47]	41.22	137.48	52.57
SegFormer [48]	23.72	163.16	54.36
Mask2Former [49]	44.01	372.84	54.63
MGA-Net [50]	40.84	186.54	68.81
IMFF-Net [41]	34.68	171.24	60.28
HiRNet (Ours)	18.92	122.96	50.27

**Table 7:** Results of the cumulative and leave-one-out ablation studies on the RVD dataset. Boldface indicates the highest values in each column. A checkmark indicates that the corresponding component is included, whereas “w/o” indicates that it is omitted. The configuration Baseline + BD + CADC + MPDT is equivalent to Full w/o HCIT and is therefore reported only once.

Variant	BD	CADC	MPDT	HCIT	SE (%)	SP (%)	ACC (%)	F1 (%)	AUC (%)
Baseline	–	–	–	–	54.31±1.20	96.54±0.34	94.43±0.26	49.37±0.59	93.77±0.42
Baseline + BD	✓	–	–	–	55.46±1.12	96.79±0.31	94.72±0.24	51.26±0.59	94.05±0.43
Baseline + BD + CADC	✓	✓	–	–	55.86±1.05	97.72±0.29	95.63±0.22	56.05±0.75	94.33±0.39
Baseline + BD + CADC + MPDT	✓	✓	✓	–	57.52±0.98	97.34±0.27	95.35±0.21	55.28±0.67	94.78±0.34
Full w/o BD	–	✓	✓	✓	56.35±1.04	97.25±0.28	95.30±0.22	52.90±0.53	94.45±0.38
Full w/o CADC	✓	–	✓	✓	55.68±1.10	96.85±0.30	94.80±0.24	51.55±0.57	94.12±0.42
Full w/o MPDT	✓	✓	–	✓	55.82±1.08	96.92±0.29	94.88±0.23	51.78±0.55	94.20±0.40
Full	✓	✓	✓	✓	58.36±0.92	97.81±0.28	95.83±0.22	58.39±0.74	95.16±0.27

**Table 8:** Cross-dataset results for DRIVE, CHASE_DB1 and RVD datasets. Boldface indicates the highest values in each column.

Method	DRIVE → RVD		CHASE_DB1 → RVD		RVD → DRIVE		RVD → CHASE_DB1	
	F1 (%)	AUC (%)	F1 (%)	AUC (%)	F1 (%)	AUC (%)	F1 (%)	AUC (%)
U-Net [13]	36.53±1.18	86.80±0.65	38.73±1.13	87.50±0.61	61.84±1.22	91.20±0.58	58.30±1.35	90.25±0.60
UNet++ [18]	37.86±1.12	87.35±0.61	39.95±1.08	88.02±0.58	63.65±1.10	92.05±0.52	60.15±1.28	91.04±0.55
AG-Net [9]	39.50±1.04	88.12±0.54	41.43±0.98	88.73±0.51	66.14±0.97	93.10±0.46	62.40±1.16	92.08±0.48
DeepLabV3+ [47]	41.11±0.97	89.04±0.49	42.84±0.93	89.61±0.46	67.44±0.91	93.65±0.43	63.50±1.09	92.65±0.44
SegFormer [48]	42.33±0.89	89.55±0.45	44.17±0.84	90.08±0.42	69.41±0.84	94.38±0.38	65.10±0.98	93.48±0.39
Mask2Former [49]	43.20±0.92	89.83±0.43	44.88±0.87	90.41±0.40	70.30±0.87	94.62±0.36	66.00±1.01	93.76±0.37
MGA-Net [50]	38.67±1.10	88.44±0.57	40.56±1.05	88.96±0.54	65.40±1.02	92.88±0.48	61.75±1.21	91.72±0.51
IMFF-Net [41]	39.92±1.03	88.76±0.53	41.76±0.98	89.27±0.49	66.89±0.95	93.42±0.44	63.10±1.12	92.40±0.46
HiRNet (Ours)	47.61±0.72	91.36±0.31	49.84±0.68	92.08±0.28	73.88±0.68	96.10±0.25	69.85±0.73	95.20±0.26

## Data Availability

The datasets used in this study (DRIVE, CHASE_DB1 and RVD) are publicly accessible via their official repositories: DRIVE (https://drive.grand-challenge.org/, accessed on 22 July 2026); CHASE_DB1 (https://www.ahajournals.org/doi/10.1161/ATVBAHA.111.225219, accessed on 22 July 2026); and RVD (https://zenodo.org/records/8287928, accessed on 22 July 2026). The implementation code and trained-model configuration used in this study are available from the corresponding author upon reasonable request.

## Abbreviations

The following abbreviations are used in this manuscript:

BD	Blur Downsampler
RVD	Retinal Vessel Dataset
HCTFE	Hybrid CNN–Transformer Feature Extractor
CADC	Consecutive Asymmetric Dilated Convolution
MPDA	Multi-Path Dilated Attention
MPDT	Multi-Path Dilated Transformer
HCIT	Hierarchical Context Information Transmission
